# Induced urinary crystal formation as an analytical strategy for the prediction and monitoring of urolithiasis and other metabolism-related disorders

**DOI:** 10.1186/1878-5085-5-13

**Published:** 2014-08-16

**Authors:** Norbert Laube, Wolfgang Berg, Falk Bernsmann, Sascha Gravius, Florian Klein, Stefan Latz, Dirk von Mallek, Tadeusz Porowski, Thomas Randau, Anna Wasilewska, Christian Fisang

**Affiliations:** 1 Deutsches Harnsteinzentrum, Urologisches Zentrum Bonn Friedensplatz, Friedensplatz 16, Bonn 53111, Germany; 2 NTTF Coatings GmbH, Maarweg 32, Rheinbreitbach 53619, Germany; 3 Clinic and Policlinic of Urology, Friedrich Schiller University of Jena, Lessingstraße 1, Jena 07743, Germany; 4 Clinic for Orthopaedics and Trauma Surgery, Friedrich-Wilhelms-Universität Bonn, Sigmund-Freud-Straße 25, Bonn 53127, Germany; 5 FH Aachen, Campus Jülich, FB Medizintechnik und Technomathematik, Heinrich-Mußmann-Straße 1, Jülich 52428, Germany; 6 Clinic and Policlinic of Urology and Pediatric Urology, Friedrich-Wilhelms-Universität Bonn, Sigmund-Freud-Straße 25, Bonn 53127, Germany; 7 Department of Research, Federal Institute for Drugs and Medical Devices (BfArM), Kurt-Georg-Kiesinger-Allee 3, Bonn, 53175, Germany; 8 Department of Pediatrics and Nephrology, Medical University of Białystok ul, Waszyngtona 17, Białystok 15-546, Poland

**Keywords:** Urinary crystal formation, Metabolic diseases, Disease/treatment monitoring, Predictive preventive personalised medicine, Bonn-Risk-Index, Nephropathies, Meno-/andropause

## Abstract

Crystal formation reflects the entire composition of the surrounding solution. In case of urolithiasis, induced crystal formation in native urine has led to the development of the Bonn-Risk-Index (BRI), a valuable tool to quantify an individual's risk of calcium oxalate urolithiasis. If the progression of a disease is associated with characteristic changes in the activities of urinary components, this leads to an altered urinary crystallisation capacity. Therefore, the results of induced urinary crystal formation can be used to detect and monitor any disease linked to the altered urinary composition. Since crystal formation inherently takes into account the entire urinary composition, the influence of the disease on individual urinary parameters does not have to be known in order to monitor the consequent pathologic alterations. In this paper, we review the background of urinary crystal formation analysis and describe its established application in urolithiasis monitoring as well as potential further fields of clinical application.

## Review

### Theoretical considerations on crystal formation *in vivo*

Crystal formation from a solution *in vivo* (in the body, temperature and pressure are considered to be constant) is governed by the system's free enthalpy *G*. The difference upon crystallisation of the free enthalpy Δ*G* of a component, which can exist in crystalline and dissolved state, can be described as [1]

(1) $$G\;=\;G_{x}\;-\;G_{s}=-\;\mathrm{RT}\;\ln\;\left(\frac{a}{a_{0}}\right)$$

with *G*_x_ and *G*_s_ as the free enthalpies of the component in the crystalline and dissolved state, respectively. *R* and *T* are the universal gas constant (8.314 JK^-1^ mol^-1^) and absolute temperature, respectively. The ratio (*a*/*a*_0_) describes the degree of saturation, whereas *a* and *a*_0_ are the component's activity in the solution and the respective activity at saturation level given by the solubility product. It is an essential condition for a crystal to form and grow that the solution is supersaturated with respect to the precipitating phases, i.e. *a*/*a*_0_ > 1. In terms of free enthalpy, supersaturation is described by

(2) G<0

the more negative Δ*G*, the higher the thermodynamic driving force for precipitation.

Non-specific ion-water and ion-ion interactions of electrostatic nature between the dissolved constituents cause ions to behave in a non-ideal manner. This non-ideal behaviour is reflected by introducing ‘activities’ instead of ‘concentrations’, whereby the activity *a*_*i*_ of a component *i* (ion or molecule) results from

(3) $$a_{i}=c_{i}\cdot \gamma_{z}$$

with *c*_*i*_ as the total molar concentration of the component *i* and *γ*_*z*_ as the mean activity coefficient for the solution's constituents with charge number *z* (*γ*_*z*_ ≤ 1 in ‘real solutions’, *γ*_*z*_ = 1 in ‘ideal solutions’). The value of *γ*_*z*_ reflects the entire interactions of an ion or molecule with all other components of the solution. Thus, *γ*_*z*_ is a measure of how much an actual system deviates from an ideal system providing the ‘effective concentration’ or ‘availability’ of a particular solute. Only for those ions analytically accessible by selective electrodes can *a* be directly determined. For all other components, *γ*_*z*_ must be approximated by empirically derived formulae, which are evaluated for different ionic strengths *I*, defined as

(4) $$I=\frac{1}{2}\sum_{1}^{i}c_{i}\cdot {z_{i}}^{2}$$

In urines, *I* typically ranges between 0.2 and 0.4 mol/l; therefore, *γ*_*z*_ can be estimated according to the Davies equation, an empirical extension of Debye-Hückel theory, which is applicable up to *I* = 0.5 mol/l [2,3]:

(5) $$\log\left(\gamma_{z}\right)=-A\cdot {z_{i}}^{2}\left(\frac{\sqrt{I}}{1+\sqrt{I}}-0.3\cdot I\right)$$

where *A* is the Debye-Hückel constant, which is a function of the temperature and dielectric constant of the solution (*A* = 0.5211 for H_2_O at *T* = 37°C). Ionic strength plays a central role in ionic activities, since in the range of validity, *γ* decreases as *I* increases.

According to the DLVO theory, ionic strength also influences the formation of agglomerates as *I* influences the thickness of the repulsive ‘diffuse electrical double layer’, which evolves from the charge of the colloid particle. At low ionic strength, the repulsive electrostatic forces of the double layer exceed the attractive Van der Waals force, and the resulting repulsion between the particles prevents them from agglomeration (unless the particle is nearly electrically neutral, which happens at the pH value of the ‘isoelectric point’). With increasing *I*, the likelihood of agglomeration increases [4].

Equations 1–5 show that crystal formation is sensitive to *any* ionic constituent coexisting in the surrounding solution. Changes in the solution's composition thus potentially take significant influence on the precipitation probability of a particular mineral phase.

### Factors affecting urinary crystal and stone formation

As described above, supersaturation is a prerequisite for the primary and secondary processes of crystal/stone formation taking place (*inter alia*, prenucleation cluster formation, nucleation, crystal growth and aggregation); its degree influences the respective process rates.

**Figure 1:**
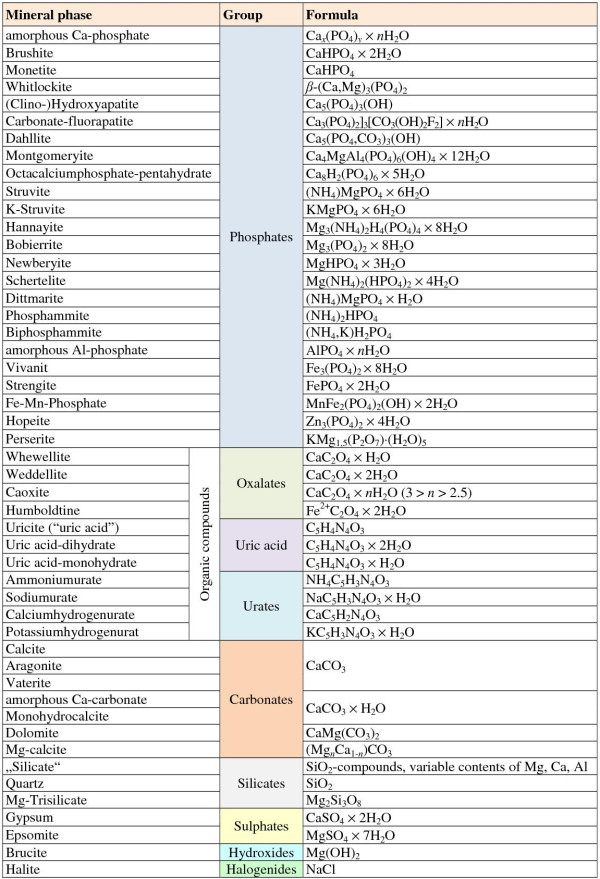
**Examples of mineral phases found in mammalian uroliths and insoluble salts precipitating from wastewater (after induction by e.g. pH rise or addition of Mg**^**2+**^**) in the course of recovery of nutrients, such as phosphorus and nitrogen.** Formulae represent ideal stoichiometric compositions. The actual compositions may differ, among others, due to partial substitution, e.g. of Ca^2+^ with Mg^2+^ or Fe^2+^ and (NH_4_)^+^ with K^+^.

**Figure 2:**
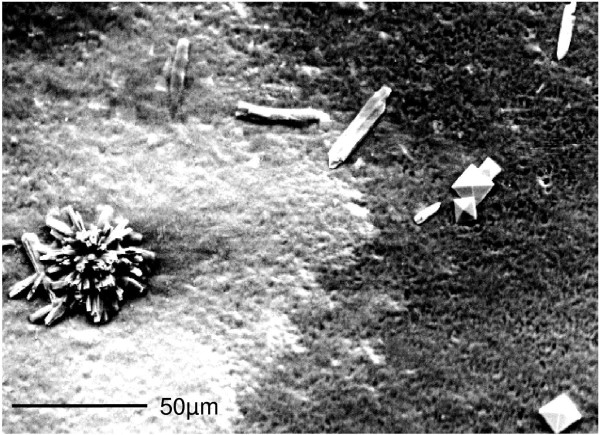
**SEM picture of urinary crystals found in the native sediment of a filtered 24-h urine of a repeatedly ‘mild hypercalciuric’ but non-stone-forming proband (pore size 0.45 μm, urinary pH = 5.81, nitrite positive, calcium excretion = 5.9 mmol/day).** Rosette-shaped calcium phosphate (left) coexists with rod-like struvite (mid) and bipyramidal calcium oxalate (right). The morphology-based analysis is confirmed by elemental analysis using SEM-linked energy-dispersive X-ray spectroscopy.

**Figure 3:**
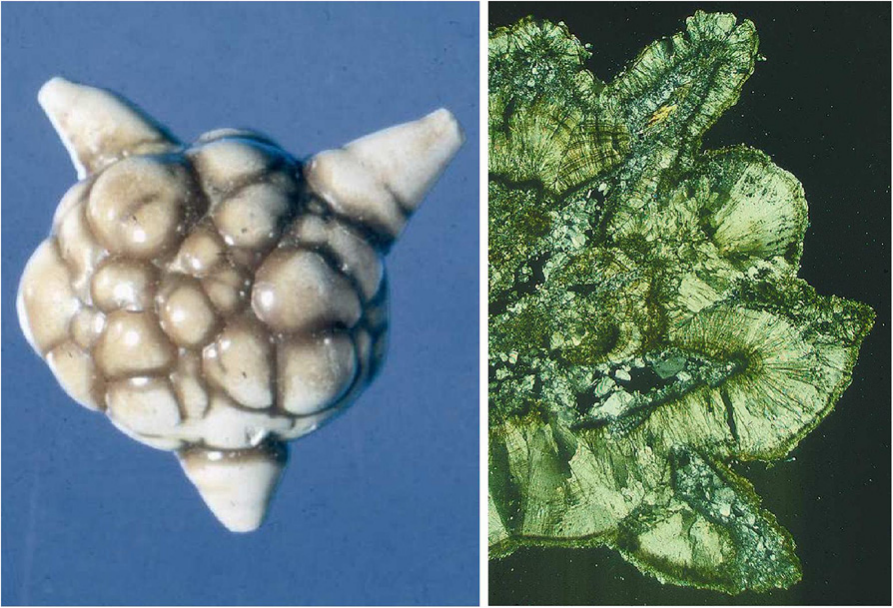
**Internal transformation processes may influence stone shape.** Left: Photograph of a mulberry**-**shaped urolith composed of whewellite with numerous facetted growth zones. Right: Thin section showing pseudomorphs of whewellite crystals in weddellite shape causing the mulberry-shaped surface of whewellite stones.

The complexity of salt formation from urine and urine-like solutions is well illustrated by the large number of different minerals found in uroliths and grown from these liquids (Figure 1) [5-14]. As the thermodynamic stability fields of these minerals partly overlap (e.g. pH range in which they show low solubility), they may form in association with others, i.e. in paragenesis (Figure 2). Around 60% of uroliths are binary or ternary mixtures of different minerals [15]. In some cases, a primarily precipitated but thermodynamically metastable mineral phase can transform (stepwise) into a more stable phase (Ostwald's rule), e.g. weddellite → whewellite (Figure 3), brushite → octacalciumphosphate pentahydrate → whitlockite → apatite and uric acid dihydrate → uric acid monohydrate.

A number of factors affect urinary supersaturation, including solute concentration and complexation, ionic strength, pH value and the presence of promoters (augmenting the rate of a chemical reaction) or inhibitors (diminishing the rate of a chemical reaction). These factors vary during the day depending on fluid intake, dietary intake and body metabolism. Urine in normal individuals is often supersaturated with respect to Ca oxalate, Ca phosphate and Na urate and has to be considered in general as a metastable solution prone to precipitate upon small changes in composition or in surface properties (e.g. suspended particles acting as crystallisation nuclei or urothelium roughness). In fact, urolith formation is strongly influenced by a multitude of different substances [16-18], e.g. acid-rich urinary proteins [19], citrate [20,21], magnesium [22], osteopontin [23-25], Tamm-Horsfall protein [26-28], polycarboxylic acids [29,30], copolymers of polyacrylic acid [31], phosphonates [32] or even ‘unidentified biomacromolecules’ [33].

Furthermore, protein concentrations (e.g. Tamm-Horsfall protein [THP], osteopontin, prothrombin fragment 1, albumin) and crystallographic variables (e.g. crystal defects, as they provide charged surface sites) may influence interfacial mineral-protein bonding and thus, e.g. by specific polyanion-crystal interactions, agglomeration of microcrystals and crystal habit [34-37].

**Figure 4:**
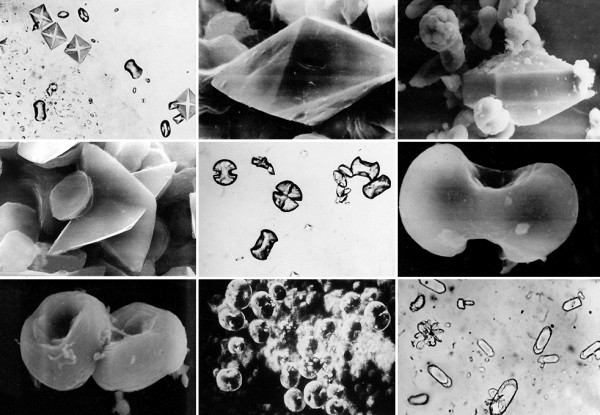
**Optical and SEM images of calcium oxalate crystals found in native urinary sediment (crystal sizes range between ≈ 20 and ≈ 35 μm).** Besides the typical whewellite crystals in egg, hour glass and barbell shapes and characteristic letter envelope shapes (tetragonal bipyramids) of weddellite crystals, numerous other crystal habits can be observed. Besides dodecahedrons of weddellite crystals, longitudinal-oval plates and mulberry habits of whewellite and twinnings also occur as well as characteristically ‘constricted’ whewellite crystals in oval shape. The simultaneous occurrence of egg, hour glass and barbell shapes at whewellite crystals can be attributed to their typically (deepened) bi-concave barbell shape. The existence of numerous habits of a mineral phase can be explained by different supersaturation conditions, diffusive processes and adsorption processes of foreign ions (e.g. [Mg^2+^], [P_2_O_7_]^4-^, [C_6_H_5_O_7_]^3-^) on preferred crystal surfaces thereby (potentially) inhibiting or promoting the growth rate of a particular crystallographic direction, which eventually leads to crystals showing different morphologies.

**Figure 5:**
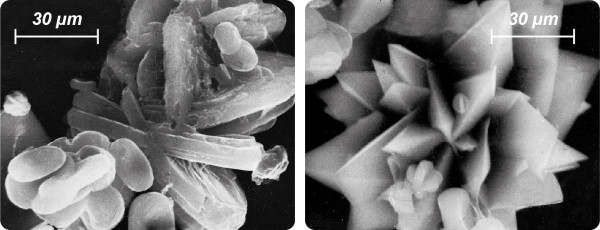
**SEM images of pathological urinary sediments of highly recurrent CaOx stone patients.** Left: Microlith composed of whewellite crystals systematically grown together (twinning). Right: Multiple interpenetrating weddellite twins indicate a lack of inhibitors of crystal growth.

The influence of minor urinary components must not be underestimated. Small concentrations of contaminants/impurities (e.g. foreign ions, mucoproteins built in the crystal structure during growth) can lead to a modified habit (Figures 4 and 5) changing the growth rate (crystal poisoning) and may have an overproportional impact on crystal formation. Inhibitors from, *inter alia*, the group of bisphosphonates (used in osteoporosis treatment), proteins and polycarboxylates can show a significant impact on crystal formation at concentrations in the order of magnitude of 1 ppm [38-41]. However, it will be impossible to determine the concentrations of all urinary constituents [42] or their particular interactions within their specific chemical formation environment.

To investigate the different mechanisms of urinary stone formation and to gain more insight into the individual role of urinary constituents with respect to supersaturation and other physicochemical considerations, numerous types of *in vitro* experiments with different approaches of chemical engineering to control, *inter alia*, the course of supersaturation are established to mimic various aspects of the (assumed) *in vivo* situation [37,43]. The more a native urine sample is supersaturated with respect to a particular mineral phase, the more easily the system can be forced to crystal formation by controlled increase of urinary supersaturation.

### Why investigating crystal formation in urine?

**Figure 6:**
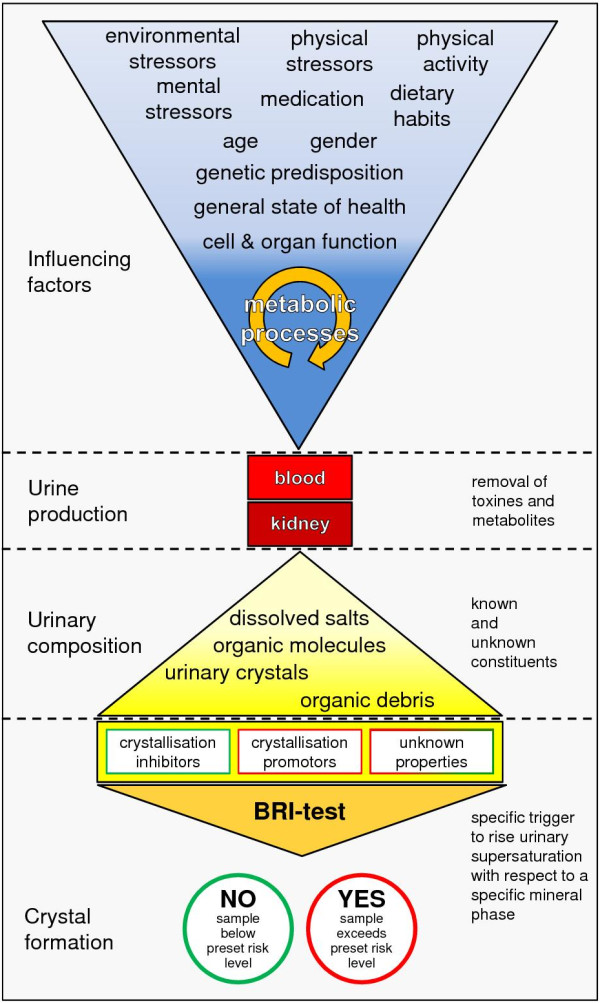
**Urine composition reflects the individual interplay of all extrinsic and intrinsic risk factors as well as all metabolic processes including affected cell and organ functions.** Depending on the medical condition, concentrations of particular urinary constituents can systematically change, and consequently, crystal formation processes are influenced. Therefore, the disease's urinary ‘signal’ can be detected by induced growth of a particular mineral phase. This relation can be used as a clinical ‘early-warning system’ if the differences in crystal formation risk and health state are analytically significant. The crystal formation risk can be detected by either continuous increase of urinary supersaturation (i.e. addition of an appropriate trigger substance) up to the moment of crystal formation (e.g. [44]) or incrementally using preset risk levels (i.e. discrete amounts of a trigger substance) that are exceeded if crystal formation takes place (e.g. [45]).

Crystal growth induced in native urine is a promising approach to quantify a disease's progression or a treatment's success. As urine composition reflects a person's metabolic state, medical conditions as well as medical treatments affecting at least one metabolic process can be detected by their specific urinary metabolic product (Figure 6). However, quantification of such a ‘biochemical signal’ by laboratory analysis, if known at all, can be expensive and time-consuming. In contrast, specifically induced formation of an appropriate mineral species from native urine skips the problem of unknown disease markers and reduces the analysis-related costs.

If a disease state is related to an altered urinary composition prone to form a particular type of crystals, the actual formation of these crystals can be easily triggered by rising supersaturation of this mineral phase by, e.g. addition of a promotoric substance to a urine sample. In a first approach, it can be assumed that the amount of added trigger substance is a function of the health state.

Therefore, induced crystal formation can be regarded as a sum parameter for ‘downstream monitoring’ of metabolic diseases. In a second step of ‘upstream analysis’, the substances assumed to significantly influence crystal formation can be identified by targeted substance-specific urinalysis.

Any individual is biologically and sociologically unique and thus reacts differently to exogenous factors in case of a disease, disorder or medical condition, in particular to therapeutic measures. It is of interest to measure the individual response in order to optimise the patient-focused treatment as well as to establish strategies for a therapeutic differentiation according to obtained patient groups. The individual reaction of a patient to a therapeutic measure affects urine composition and thus is integrally included in the crystal formation behaviour. By patient stratification, associations and interactions of risk factors as well as of anamnestic and diagnostic information can be evaluated for discrimination of therapy responder vs. non-responder. In contrast, evaluation of only a few selected primary markers and secondary control parameters of metabolic diseases (and occasionally combining them to risk indices) is often ineffective, complicated, costly and time-consuming.

### Risk indices

Risk indices are indicators which facilitate the choice of an appropriate therapy for the individual patient based on laboratory data and epidemiological observations. A risk index classifies the health status of the examined person with respect to the investigated disease on a standardised scale. It can be translated into an individual risk for the person to contract the disease. This way, a risk index transforms an otherwise unmanageable amount of data into useful information for decision-making. Risk indices can be used to characterise a patient's base line risk and to monitor the success of the prescribed treatment scheme.

### Risk indices in urolithiasis

Patients developing a disease despite the fact that all of their individual laboratory parameters lie in their respective normal range are a common challenge in everyday practice. Most stone formers are idiopathic, i.e. there is no single quantity indicating their disease, because stone formation is mainly caused by a complex imbalance between all promoting and inhibiting urinary factors [46,47].

In these cases, combination of the excretion values of several lithogenic and other promotory and inhibitory substances and of further urinary characteristics, such as 24-h volume and pH value, to multiparameter ratios or other mathematical expressions, can improve the diagnostic information and facilitate therapeutic decision-making. As these approaches account for the antagonistic processes involved in urinary stone formation, they promise better distinction between stone formers or persons prone to form stones on the one hand and normal subjects or successfully treated former stone patients on the other hand.

Based on urinalysis parameters, simple quotients with promotory variables in the numerator and inhibitory ones in the denominator are calculated. A variety of such indices exists [48-51]; examples are the concentration ratios of calcium, citric acid, oxalic acid and magnesium [Ca]/[CA] and ([Ca] [OA])/([CA] [Mg]).

A prominent example of a more complex risk index for calcium oxalate, the most common mineral in urinary stones [52], is the AP(CaOx) index. Tiselius developed it as an analytical, non-iterative approach to approximate the ion-activity product with respect to calcium oxalate from urinalysis data [53]. AP(CaOx) combines the most influencing parameters into a quotient according to the results obtained from the calculations [54]:

(6) $$\mathrm{AP}\left(\mathrm{CaOx}\right)=1.9\;\frac{{\left\{\mathrm{Ca}\right\}}^{0.84}\;\left\{\mathrm{OA}\right\}}{{\left\{\mathrm{CA}\right\}}^{0.22}{\left\{\mathrm{Mg}\right\}}^{0.12}V^{1.03}}$$

with {Ca}, {OA}, {CA}, {Mg} and *V* as the urinary 24-h excretions of calcium, oxalic acid, citric acid and magnesium and the 24-h urine volume, respectively. The different exponents in Equation 6 reflect the different influences of each of the quantities on the ion-activity product and thus on calcium oxalate formation. The exponents as well as the prefactor were continuously adjusted to improve the approximation to the updated calculations of ion-activity products.

However, despite the manageable number of laboratory parameters and the use of excretion values (instead of the thermodynamically more reasonable concentration values) as commonly accepted by clinicians for evaluation of the patient's metabolic state, AP(CaOx) is often considered too ‘cryptic’ and thus only rarely applied.

Another type of risk indices is based on computer simulations of commonly accepted thermodynamic equilibrium models of complex chemical interactions in urine, taking into account the most important urinary components. The simulations iteratively calculate the ion activities and the concentrations of potentially formed coexisting complexes from an initial chemical analysis of several urinary components (e.g. [H_3_O^+^], [Na^+^], [K^+^], [Ca^2+^], [Mg^2+^], [NH_4_^+^], [SO_4_^2-^], [PO_4_^3-^], [CA], [OA]) and finally the relative supersaturations and saturation index for (all) potentially precipitating salts. Computer programs providing these features include EQUIL [55,56] and JESS [57-59].

Although these classic thermodynamic approaches promise the best results for estimation of urinary supersaturation from urinalysis by including a large number of urinary components (23 at the most enhanced program version, EQUIL93 [55]), it seems that this variety of input variables and the efforts and time required to obtain the result hinder, like for AP(CaOx), a more widespread use of the EQUIL and JESS tools.

#### The Bonn-Risk-Index-approach

Salt-forming components of naturally occurring uroliths are analytically accessible, and empirically derived algorithms (see above) were developed allowing for calculation of the activity product to estimate the urolithiasis risk. However, similar urinary compositions with respect to major components can show quite different pathological patterns *in vivo*. Furthermore, about 70% of stone formers are considered idiopathic, i.e. metabolic diseases like renal tubular acidosis or hyperparathyroidism as well as anatomical causes, like ureteral stenosis, can be excluded and urinalysis does not show (significant) abnormalities.

Unidentified but influencing constituents and unpredictable ‘Dr. Jekyll and Mr. Hyde’ behaviour of known substances complicate the evaluation of a ‘real’ crystallisation risk even on the basis of the most detailed urinalysis. In particular, the role of (intra-crystalline) macromolecules (mainly proteins, also lipids and carbohydrates), which form an organic matrix in uroliths comprising in total 2%–10% of the stone's total weight, remains disputable as they may be either inhibitors or promoters or ‘bystanders’ of stone formation [60-63]. In fact, the physicochemical properties (e.g. protein structure, ionic charge) and therefore the effects of a particular urinary constituent are dependent on the entire chemical environment (e.g. ionic strength).

To overcome the drawbacks of analyses based on selected urinary parameters, a non-specific test was developed including all urinary constituents to an effect-directed (‘downstream’) analysis by controlled induction of urinary calcium oxalate crystal formation combined with an individual test of an ‘unequivocally crucial and easy-to-determine’ single parameter, the concentration of ionised calcium [Ca^2+^]. The amount of ammonium oxalate (Ox^2-^) required to induce formation of CaOx crystals (originally) in a 200-ml sample of native 24-h urine or 2 × 12-h urine (e.g. comprising a night and a day fraction, each lasting 12 h) and [Ca^2+^] are used to calculate the BRI [44]:

(7) $$\mathrm{BRI}=\frac{\left[{\mathrm{Ca}}^{2+}\right]}{\left({\mathrm{Ox}}^{2-}\right)}$$

A scale of eight BRI levels was developed to determine the risk of CaOx urolithiasis [64]. The BRI shows superior diagnostic sensitivity and specificity compared to other urolithiasis risk indices [65].

Many diseases are lacking a simple, easily accessible and cost-saving diagnostic approach to routinely monitor the disease's path and treatment success. Since crystal formation in general and the BRI in particular take into account every constituent of native urine, they can be used to monitor a wide range of metabolic diseases. If the BRI concept is adapted to a particular disease, it can be used for a fast initial diagnosis before the beginning of therapy and for accompanying diagnostics for decision support to find a patient-tailored therapy.

The BRI can be determined either manually in a biochemical laboratory or in a fully automated benchtop measuring device, the Urolizer®. Currently, a microfluidic device to estimate the BRI (BRI-on-Chip) is under development [45]. Different reaction channels containing appropriate chemicals will indicate the BRI class of a small urine sample as ‘low’, ‘medium’ or ‘high’ risk of calcium oxalate urolithiasis. The result will be displayed by a colour reaction to be read out by the naked eye or using a portable (true point-of-care) diagnostic tool like, for example, a smartphone app for quantification [66,67]. Therefore, the BRI-on-Chip will take BRI determination from specialised laboratories to the resident doctor's cabinet or even to the patient's home upon the doctor's advice.

This way, the effects of, for example, different dietary concepts like the actual influence of e.g. sodium chloride, protein and calcium intake on an individual's urinary Ca excretion and the effect of counter measurements can be tested by the patient on an individual level under real-world physiological conditions (everyday activities, travel, exercise and food consumption) in due time.

#### Application potential

##### Urolithiasis

**Figure 7:**
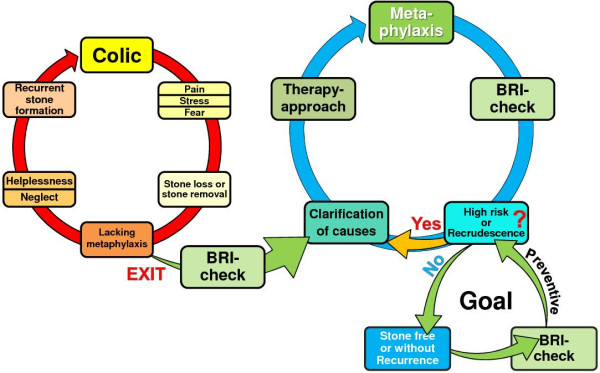
**The role of the BRI-test in the prevention of recurrent stone formation.** Left: The vicious circle of recurrent stone formation as a result of lacking metaphylactic measures. Right: The BRI has been proven to be a straightforward and intuitive diagnostic tool helping to escape from therapeutic undersupply and to achieve health and therapy control.

The BRI has proven its qualities as a diagnostic tool in the treatment of calcium oxalate urolithiasis. Urolithiasis is a widespread ailment in developed countries with current prevalence rates above 5%. Stone formation is the result of an altered urinary composition, mostly caused by a congenital or acquired underlying metabolic disease (or at least a predisposition to it) and by triggering exogenous risk factors. This makes the pathogenesis in each patient unique and diagnosis, causal treatment and therapeutic monitoring difficult, costly and time-consuming. Routine diagnostic workup is characterised by a lack of analytical results easy to interpret for clinical decision-making and by weak financial incentives. The consequence is a diagnostic and therapeutic undersupply, which inevitably results in a vicious circle of recurrent stone formation, although the recurrence rate can be lowered from 80% without metaphylaxis down to 20% under risk-adopted therapy (Figure 7).

**Figure 8:**
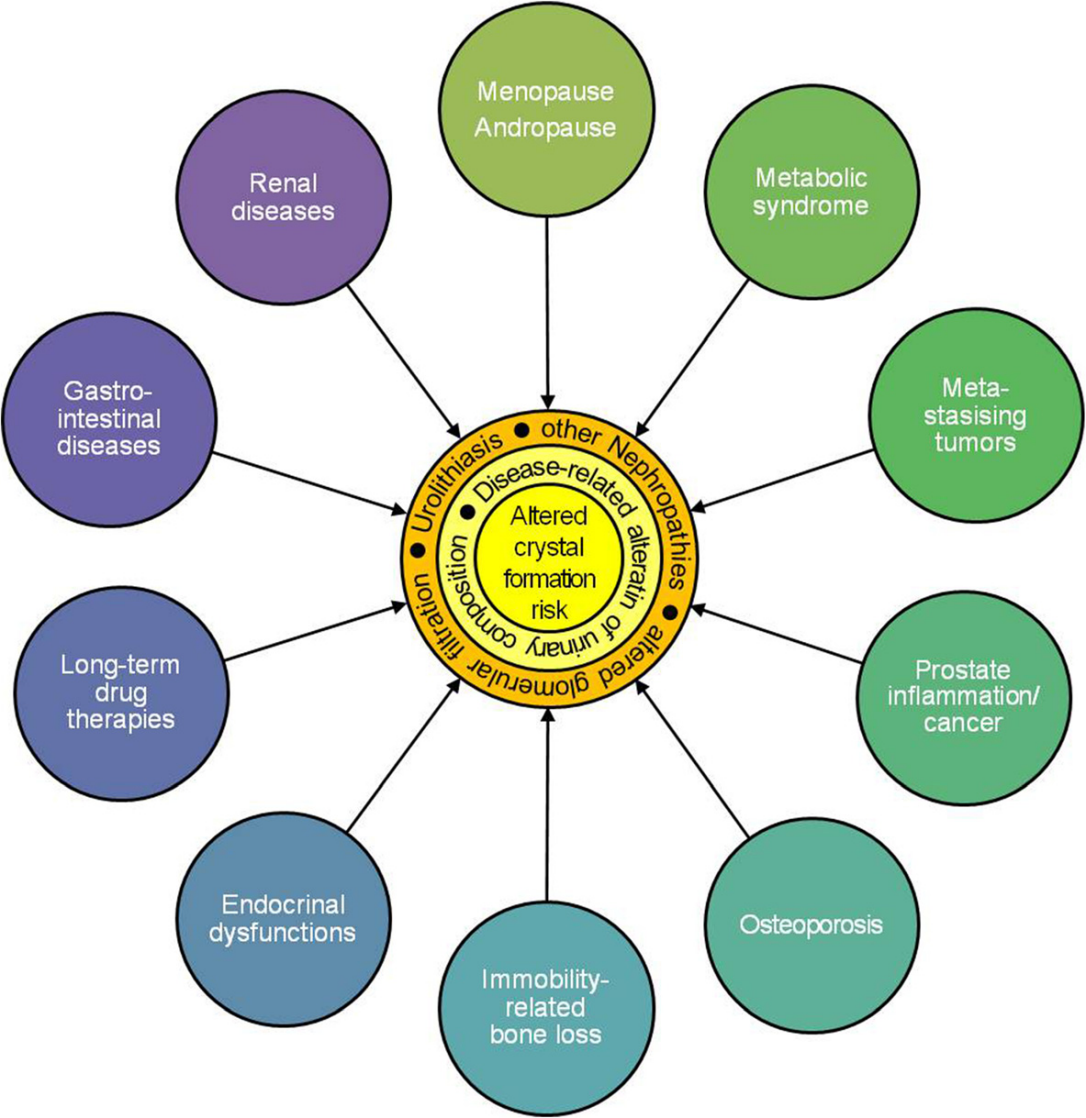
**Examples of disease patterns affecting renal function or urinary composition.** The resulting urine's crystal formation risk with respect to a particular salt can be specifically and significantly altered compared to that of a healthy subject.

A strategic shift from ‘disease care’ (i.e. repeated cost-intensive and side effect beset ‘stone removal only’) to ‘health care’ by individualised recurrence prevention could be achieved by providing adequate diagnostic tools. A point-of-care device like the BRI-on-Chip to measure urolithiasis risk by induced urinary crystal formation might be used for screening (for prophylaxis, e.g. children with a parent suffering from recurrent urolithiasis or chronic renal disease) and as a test for individual adjustment and effective control of metaphylaxis.Induced crystal growth in urine can be a powerful strategy not only in urolithiasis therapy but also for a wide spectrum of diseases affecting metabolism (Figure 8), either to identify chronically altered or changing renal clearance patterns associated with metabolic diseases, or for targeted evaluation of the overall influence of therapeutic measures, including dietary habits (i.e. treatment success). In the following paragraphs, some diseases known to affect renal handling and to augment crystal formation risk are presented, which might benefit from BRI-like analyses of induced urinary crystal formation.

##### Osteoporosis

Bone constitutes the largest depot of calcium in the human body. Osteoporosis describes the loss of bone mineral density and is associated with an increased risk of fractures, especially in the spine, hip and wrist. The decrease in bone mineral density is a hormonally regulated, mostly age-related process. Typically, postmenopausal women are prone to suffer from the disease, but other reasons like inactivity, medication or hormonal dysregulation can also be associated with osteoporosis. While degradation products of bone resorption, such as calcium and the c- and n-terminal collagen crosslinks, are currently used as indicators for osteoporosis, neither elevated calcium levels nor the presence of c- and n-terminal collagen crosslinks (CTX/NTX) in urine is a sufficiently sensitive diagnostic parameter. Furthermore, measurement of CTX/NTX is only available in specialised laboratories. We assume that changes in bone turnover due to osteoporosis and other bone-related diseases alter the urinary stone formation risk. Thus, it would be possible to correlate BRI results with the results of bone mineral density scans, bone turnover markers and fracture rate to determine its value in predicting and monitoring increased bone resorption and success of the medical treatment.

##### Risk of immobility*-*related changes in metabolism and bone loss

A significantly increasing BRI-based crystallisation risk, e.g. of calcium oxalate or uric acid dihydrate, while bedridden would allow for early identification of patients prone to form osteoporotic and nephropathic disease patterns and timely initiation of preventive measures (e.g. Ca supplementation, vitamin D, allopurinol). The individual effect of prevention or countermeasures can be evaluated as at ‘overdosage’, and the risk of calcium oxalate crystal formation concomitantly increases again. Therefore, the individual Ca requirement can be evaluated.

##### Metastatic malignancies

Bone metastases from solid tumours (e.g. prostate cancer) might be detected earlier if urinary crystal formation is regularly used to monitor short-term changes in bone mineral balance. Few studies are concerned with urinary calculi in malignancies. Assumedly, there is a correlation due to an increase of crystallisation promoters by metabolic/paraneoplastic factors or elevated bone turnover following low bone density or bone metastases. (Hyper-)calciuria, a common promoting factor of urinary calculi, was described to be a diagnostic criterion in hormonal therapy of metastasising breast carcinoma. Hypercalciuria was also found in adenocarcinoma of the lung. Without reference to metabolic or laboratory details, the association between some malignancies and urinary calculi is described, e.g. in kidney cancer, squamous cell carcinoma of the urinary tract or ameloblastoma [68-72]. Gawade et al. [73] described older survivors of childhood acute lymphoblastic leukaemia with low bone density to be at risk for renal stones. Camacho et al. [74] observed hypercalciuria as a secondary cause of bone loss in 15.6% of breast cancer patients.

##### Diabetes mellitus

A premature detection of early stages of nephropathy development might be achieved due to a rise in glomerular loss of macromolecular blood components (e.g. impaired creatinine clearance, proteinuria [e.g. albumin, THP]) and altered biomarker patterns related to a progressive course of renal insufficiency. The correlation between urinary crystal formation risk and potential development of secondary complications or co-morbidities in patients diagnosed with DM should be investigated. Progressive diabetic nephropathy is characteristic in about 60% of DM patients. Complementary to the BRI technology, blood samples should be investigated for potentially increased metalloproteinase activity as an independent biomarker for tissue remodelling which accompanies a development of diabetic complications (proliferative retino- and nephropathy). An increased predisposition to urinary stone formation and extensive tissue remodelling are considered as the optimal biomarker panels for early prediction of severe DM complications.

##### Nephropathies

No other organ is more involved in metabolic processes than the kidney. Thus, it is no surprise that (non-diabetes-related) nephropathies [75] go along with metabolic disorders, partially with an increase of crystallisation promoters or, more generally, nephropathy-related specific changes in urinary composition. For example, hypercalciuria was described in Bartter's syndrome or renal sarcoidosis. Minimal change in nephrotic syndrome can result in focal calcification and hypercalciuria [76-78]. Praga et al. [79] found a high prevalence of hypercalciuria and nephrolithiasis in patients with thin basement membrane nephropathy.

##### Prevention of drug-induced crystal nephropathies

Chronic intake of xenobiotics including a wide variety of drugs can have side effects which result in specific alterations of urinary composition. These alterations may cause a significantly increased risk of abnormal crystalluria (syn. microlithiasis) and urolith formation by, *inter alia*, drug-induced hypercalciuria (e.g. loop diuretics, glucocorticoids, vitamin D, calcium supplementation), hyperoxaluria (e.g. antibiotics, pyridoxilate), hyperuricosuria (e.g. uricosurics, chemotherapeutic agents) or unfavourable changes in urinary pH value (e.g. alkali citrates, Na bicarbonate, antacids). Depending on dose and duration of treatment, oxalates, Ca phosphates, uric acid dihydrate or urates can precipitate. Furthermore, the non-metabolised drugs themselves or their less-soluble metabolites can form urinary crystals (e.g. protease inhibitors) [80-84]. Patients, requiring (high-dose) long-term treatment with potentially nephropathy-inducing drugs, thus may profit from early recognition of an increased likelihood of intra-renal crystal precipitation by regular easy-to-perform monitoring of their urolithiasis risk. At significant changes in that parameter, prophylaxis and metaphylaxis measurements (e.g. control of individual drug response and dose effects, dosage adjustment, pH adjustment, adequate hydration) can be initiated at a very early stage.

##### Therapeutic drug monitoring

Medications with a narrow therapeutic range and for which target concentrations are difficult to monitor (i.e. critical dose drugs, e.g. Aciclovir, Digoxin, Lithium, Methotrexate) have to be carefully supervised [85]. The more the patient shows risk factors (e.g. age, body mass index, organ function, concomitant drug therapy) and morbidities (e.g. hepatic diseases, nephropathies, cardiac insufficiencies, diabetes mellitus), the more important is the finding and dynamic adjustment of the optimal, i.e. individual, dosage regimen. Investigation of urinary crystallisation risk targeting on the drug itself or at a specific drug-related metabolite instead of e.g. analysis of plasma drug concentration can be a cost-effective alternative.

##### Outlook

The approach introduced above may serve as the ‘proof-of-principle’ technological platform for a broad spectrum of clinical applications. By development of a simple crystallisation test device (BRI-on-Chip) with one individual indication-adopted preset ‘cut-off’ value, or with multiple graduated cut-off values combined in one set, the analysis of induced urinary crystallisation can be taken from specialised laboratories to the doctor's office or even the patient's home.

In the long term, the analytical method of induced crystal growth might be transferred to other body liquids like cerebrospinal fluid or synovial fluid, thereby opening further application fields for the BRI approach.

For the practical realisation of the approach, a series of large-scaled multidisciplinary studies might be useful for international validation and clinical adaptation. In order to create the appropriate international partnerships within the field, the new European programme ‘Horizon 2020’ may be considered. The complete overview of the strategies and instruments of Horizon 2020 is provided by the ‘Predictive, preventive and personalised medicine as the hardcore of “HORIZON 2020”: EPMA position paper’ [86].

## Conclusions

The BRI-on-Chip rapid test under development can be an effective screening and diagnostic tool for many metabolic diseases by induced urinary crystallisation. As any urinary constituent influences the proneness to crystal formation (and the characteristics of the minerals developed), any change in urine composition leading to a variation in BRI can indicate a change in the patient's general metabolic state or disease condition. Therefore, the BRI can facilitate finding therapies targeted to the individual patient's metabolism.

Analysis of difficult-to-obtain or expensively-to-determine biochemical parameters may be avoided if a clinically relevant correlation between BRI and the course of a disease can be established. Clinical studies to systematically investigate BRI, e.g. in patients with bone diseases like osteoporosis, are planned.

Urinary crystal formation within a point-of-care test represents a strategy for rapid (downstream) analysis to detect early stages or progression of metabolic diseases. If necessary, detailed analysis of (cost- and time-intensive) individual parameters can be performed in a second step to complement the BRI-based diagnosis.

The proposed strategy to analyse a person's state in respect to a particular metabolic disease by specific investigation of his/her urine's tendency to form crystals by forced growth of a mineral species can be termed ‘urinalytiXX’, derived from ‘urine analytics by crystallisation’, where ‘XX’ stands for ‘crystallisation’—an often used abbreviation in the field of geosciences.

## Abbreviations

BRI: Bonn-Risk-Index; CA: citric acid; CaOx: calcium oxalate; CTX/NTX: c- and n-terminal collagen crosslinks; DM: diabetes mellitus; OA: oxalic acid; SEM: scanning electron microscopy; THP: Tamm-Horsfall protein.

## Competing interests

FB is an employee of NTTF Coatings GmbH, a company involved in the development of the BRI-on-Chip. NL is the inventor of a patent on the BRI-on-Chip concept. The other authors declare to have no competing interests.

## Authors' contributions

NL developed the BRI, conceived to extend the BRI approach to other diseases and did the organisation of the study and article preparation. WB contributed information on the mineral phases and phase transitions observed in uroliths. FB coordinated the scientific team, participated in the design and coordination of the study and contributed to the drafting, writing and reviewing of the manuscript with focus on chemistry. FK performed literature search in the field of waste water technology and supported the article preparation. SG, SL, DvM, TP, TR, AW and CF contributed to the drafting, writing and reviewing of the manuscript. CF coordinated the urology part, performed literature search and drafted the article with focus on urology. All authors read and approved the final manuscript.
